# A review of the direct targets of the cannabinoids cannabidiol, Δ9-tetrahydrocannabinol, N-arachidonoylethanolamine and 2-arachidonoylglycerol

**DOI:** 10.3934/Neuroscience.2024009

**Published:** 2024-04-30

**Authors:** Nicholas J. D. Wright

**Affiliations:** Wingate University School of Pharmacy, Wingate, North Carolina 28174, USA

**Keywords:** Cannabidiol, Δ9-tetrahydrocannabinol, N-arachidonoylethanolamine, 2-arachidonoylglycerol, endocannabinoid system

## Abstract

Marijuana has been used by humans for thousands of years for both medicinal and recreational purposes. This included the treatment of pain, inflammation, seizures, and nausea. In the 1960s, the structure of the principal psychoactive ingredient Δ9-tetrahydrocannabinol was determined, and over the next few decades, two cannabinoid receptors were characterized along with the human endocannabinoid system and what it affects. This includes metabolism, the cardiovascular and reproductive systems, and it is involved in such conditions as inflammation, cancer, glaucoma, and liver and musculoskeletal disorders. In the central nervous system, the endocannabinoid system has been linked to appetite, learning, memory, and conditions such as depression, anxiety, schizophrenia, stroke, multiple sclerosis, neurodegeneration, addiction, and epilepsy. It was the profound effectiveness of cannabidiol, a non-psychoactive ingredient of marijuana, to relieve the symptoms of Dravet syndrome, a severe form of childhood epilepsy, that recently helped spur marijuana research. This has helped substantially to change society's attitude towards this potential source of useful drugs. However, research has also revealed that the actions of endocannabinoids, such as anandamide and 2-arachidonoylglycerol, and the phytocannabinoids, tetrahydrocannabinol and cannabidiol, were not just due to interactions with the two cannabinoid receptors but by acting directly on many other targets including various G-protein receptors and cation channels, such as the transient receptor potential channels for example. This mini-review attempts to survey the effects of these 4 important cannabinoids on these currently identified targets.

## 1. Introduction

**Figure 1.**
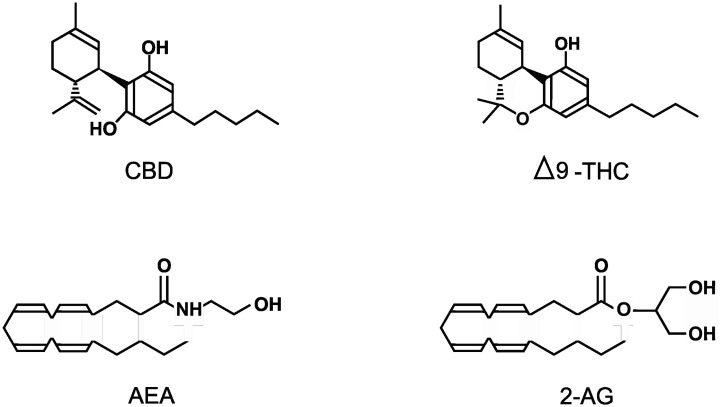
Representations of the structures of the 4 cannabinoids discussed in this review including Cannabidiol (CBD), Δ9-Tetrahydrocannabinol (THC), N-Arachidonoylethanolamine (AEA) and 2-Arachidonoylglycerol (2-AG).

The cannabis plant, more commonly known as marijuana, has been used by humans for thousands of years but only recently has the cannabis genus (which includes the species *Cannabis sativa, Cannabis indica*, and *Cannabis ruderalis*) been investigated for its pharmacological potential. The first recorded medical use was in China over 5,000 years ago, where marijuana was used to treat pain, inflammation, seizures, and nausea. But it was also used recreationally and it was this use, especially in the West, that prevented its full medical potential from being explored [1],[2]. Nearly 60 years ago, Δ9-tetrahydrocannabinol (THC; see Figure 1) was isolated along with many other phytocannabinoids from marijuana [3]; this made possible the identification and characterization of the first endogenous cannabinoid receptor (CB1R) which is a G-protein coupled receptor (GPCR) [4],[5]. This was soon followed by the identification of a second endogenous cannabinoid receptor (CB2R; also a GPCR) [6],[7]. The expression of these 2 cannabinoid receptors, along with the presence of the principal endogenous endocannabinoids N-arachidonoylethanolamine (AEA: anandamide; see figure 1) and 2-arachidonoylglycerol (2-AG: see figure 1) helped define the endocannabinoid system [8]–[11]. The endocannabinoid system in the central nervous system (CNS) can affect appetite, learning, memory, and conditions such as depression, anxiety, schizophrenia, stroke, multiple sclerosis, neurodegeneration, addiction, and epilepsy [1],[10],[12],[13]. In the peripheral nervous system and other tissues this system can affect metabolism, nociception, the cardiovascular and reproductive systems, and conditions such as inflammation, cancer, glaucoma, and liver and musculoskeletal disorders [14],[15]. However, over the last couple of decades it became clear that the many actions of cannabinoids could not be just attributed to interactions with the two cannabinoid receptors. Now it is known that cannabinoids can interact with a wide variety of additional targets ranging from glycine receptors to opioid receptors to various transient receptor potential channels (TRPs). The purpose of this mini-review therefore is to summarize the current known direct targets of four important cannabinoids including cannabidiol (CBD; see Figure 1), THC, AEA, and 2-AG; the chemical structures of these cannabinoids are illustrated in Figure 1. In order to be included in this mini-review, the authors had to be able to state with a high degree of certainty that the cannabinoids were acting on the specific target and not via another pathway or receptor. This mini-review does not cover any transporter, metabolic, or indirect targets. No actions of metabolites or synthetic cannabinoids are discussed and the reader is referred to more specific reviews on these subjects.

## 2. The agonists

CBD was first isolated from marijuana in 1940 by Adams et al. and from hashish resin by Jacob and Todd [16],[17], and its structure was later elucidated in the 1960's by Mechoulam and Shvo [18]. In 1964, Gaoni and Mechoulam described the structure of the psychoactive phytocannabinoid THC [3]. It is interesting to note that although CBD and THC are considered the major active phytochemicals in marijuana they are not thought to be normally synthesized in the plant but rather produced by heat-induced decarboxylation of other related phytocannabinoids such as cannabidiolic acid [19],[20]. The endogenous cannabinoids AEA and 2-AG are arachidonic acid derivatives formed from the cell membrane components N-arachidonoyl phosphatidylethanolamine and diacylglycerol [21]. AEA was the first endocannabinoid identified that acted on cannabinoid receptors and was described in 1992 [8] while 3 years later 2-AG was also identified [9].

## 3. Cannabinoid targets

### 3.1. The cannabinoid receptors CB1R and CB2R

CB1R is a 472 amino acid GPCR in humans which has been characterized [4],[5],[22]–[24] together with 3 isoforms that exhibit high levels of expression [25] throughout the CNS in areas such as the olfactory bulb, hippocampus, basal ganglia, and cerebellum and peripherally in sympathetic terminals, trigeminal ganglion, dorsal root ganglia, and nociceptive terminals [26]–[29]. As a GPCR, this receptor acts via the heterotrimeric G-protein G_i/o_ (α-subunit) to inhibit adenylate cyclase (AC) and therefore lower the concentration of cyclic adenosine monophosphate (cAMP) in the cell [30]. It is thought that the β/γ-heterodimer can itself activate some AC isoforms [31],[32] and CB1R may also act via G_q/11_ to increase intracellular calcium [33]. This receptor has also been reported to directly interact with certain calcium channels [34],[35] and potassium channels [36],[37] and may activate cell survival pathways [38],[39]. CB2R is a 360 amino acid GPCR in humans which is found as 2 differentially expressed isoforms [6],[7],[40]. This cannabinoid receptor is expressed primarily outside the CNS in peripheral tissues such as the immune, cardiovascular, reproductive, and gastrointestinal systems [6],[30]. This GPCR can also inhibit AC and therefore decrease intracellular cAMP, activate the mitogen-activated protein kinase (MAPK) pathway affecting gene expression [41],[42], and promote neuronal survival similarly to the CB1R [43]. Now, examining the actions of the 4 selected cannabinoids, CBD appeared to have little effect on receptor activation and may act as an antagonist on both receptors [44]–[46]. In fact, CBD can negatively modulate the responses to THC and 2-AG [47],[48]. In 2019, Tham et al. demonstrated CBD acting as a negative allosteric regulator on CB1R and as a partial agonist on CB2R in HEK293A cells [49]. THC can act as a partial agonist on both cannabinoid receptors [30],[50],[51]. AEA is a high affinity partial agonist of CB1R but appears virtually inactive on CB2R [52] whereas 2-AG only exhibits moderate affinity as a partial agonist for both cannabinoid receptors [7].

### 3.2. G-protein coupled receptors 3, 6, 12, 18, and 55

The current unnamed (orphan) GPCRs 3, 6, and 12 only appear to have been explored in their responses to CBD which acts as an inverse agonist for all 3 [53]–[55]. GPCR18 was isolated in 1997 by Gantz et al. and is typically coupled to G_i/o_ which inhibits cAMP production and is expressed at high levels in the brainstem, spleen, and testes [56],[57]. CBD appears to have little effect on this receptor at physiological concentrations [58] whereas THC is a potent agonist in HEC-1B cells expressing this receptor [59]. AEA and 2-AG seem to have little effect on GPCR18 although McHugh reported that AEA could act as an agonist in HEC-1B cells expressing this receptor [59]. GCPR55 was characterized by Ryberg et al. in 2007 and is highly expressed in the CNS, adrenal glands, and gastrointestinal tract, and it acts primarily through activation of G_13_ [60],[61]. Using HEK293 cells, this GPCR was also observed to couple to Gq [62]. CBD acts as an antagonist on this receptor [63] whereas Ryberg using HEK293 cells showed THC can be an agonist [61]. AEA may act as a partial agonist but 2-AG had little effect using an assay which observed increases in intracellular calcium in HEK293 cells and also in U2OS cells as evidence for GPCR55 activation [62],[64].

### 3.3. Opioid receptors µ, δ and κ

All opioid GPCRs couple principally to Gi/o and in most neurons reduce excitation by increasing potassium currents. In addition to inhibiting AC they activate MAPK [65]. It had been discovered in the 1970s that THC could reduce the symptoms of naloxone-induced opioid withdrawal due to interactions with the µ receptor [66],[67]. Labelled THC binding was observed at µ opioid receptors in rat brain [68],[69] and it was found both THC and CBD allosterically modulate both µ and δ opioid GPCRs negatively [70] while THC alone appears to interact with κ opioid receptors [71]. AEA and 2-AG had no apparent direct effect on opioid receptors, although there appears to be mounting evidence that cannabinoid receptors may directly interact with opioid receptors [72],[73].

### 3.4. Purine and adenosine receptors

Apart from being central in the energetic status of a cell, purines are involved in a wide range of physiological functions [74],[75]; adenosine receptors are part of the purine family. Adenosine receptors are GPCRs that typically couple to G_s_ or G_i/o_ to activate or inhibit AC, respectively. They can also interact with calcium and potassium channels via the activated G-proteins. These receptors used to be known as P1 receptors but now they have been renamed after their primary ligand adenosine receptors A_1_, A_2A_, A_2B_, and A_3_ [74],[76]. CBD allosterically enhances adenosine receptors A_1_ and A_2A_ [77],[78] while 2-AG was found to allosterically inhibit A_3_ adenosine receptors [79]. THC and AEA appear not to have any actions on these receptors.

### 3.5. Peroxisome proliferator-activated receptors (PPARα and PPARγ)

PPARs are ligand-activated transcription factors that are members of the nuclear hormone receptor family and which are important in regulating energy and metabolic homeostasis; in particular they are involved in insulin sensitivity and enhancing both glucose and fatty acid metabolism [80]. It has been shown that CBD and THC can activate PPARγ [81]–[84] utilizing fatty acid binding proteins to transport them into the nucleus [85]. However, Alhamoruni saw little direct effect with either CBD or THC in Caco-2 cell cultures [86],[87]. Apparently, AEA and 2-AG can activate both PPARα and PPARγ [88]–[91].

### 3.6. Glycine receptor

The ionotropic glycine receptor is a pentameric ligand-gated chloride channel which mediates neuronal inhibition both in the brain stem and spinal cord [92]. It belongs to the Cys-loop ionotropic family of receptors which also includes nicotinic acetylcholine, serotonin type 3, and GABA_A_ receptors [93],[94]. The effect of cannabinoids on these receptors appears to be subunit-dependent similarly to other such receptors in this family as discussed later. CBD and THC were found to potentiate glycine's actions in mice and this effect required certain alpha subunits (α-1, α-1-β, α–2 and α-3 subunits) in the receptor [95],[96]. Hejazi et al. found that THC and AEA also potentiated glycine currents allosterically in Xenopus oocytes expressing glycine receptors [97]. However, Lozovaya et al. found in isolated rat hippocampal neurons and cerebellar Purkinje neurons that AEA and 2-AG inhibited glycine receptors and accelerated desensitization [98]. In HEK293 cells expressing glycine receptors of varying subunit composition AEA potentiated the effects of these receptors if containing α-1 or α-1-β subunits but had no effect on receptors made up of α-2 or α-3 subunits [99]. Alvarez et al. recently published a paper using computer modelling, among other methods, to investigate the molecular mechanisms possibly involved in the positive allosteric modulation of glycine receptors by THC [100].

### 3.7. Nicotinic receptor

These ionotropic pentameric acetylcholine receptors that mediate excitation by sodium ion entry are found throughout the body specifically at autonomic ganglia and neuromuscular junctions (NMJ). In the CNS they are widespread and are typically found on presynaptic terminals where they can enhance or inhibit the release of other neurotransmitters. Additionally, their responses to cannabinoids are subunit dependent [101],[102] as seen with glycine receptors that are also members of the Cys-loop family. CBD has been shown to inhibit synaptic transmission at frog NMJs, human nicotinic receptors, and rat hippocampal neurons whereas THC appears to have little effect [103]–[105]. AEA and 2-AG antagonize nicotinic receptors in Xenopus oocytes [103],[106],[107]. Cannabinoids seem most effective on these receptors if homologous for α7 subunits or α4β2 combinations [108].

### 3.8. 5-Hydroxytryptamine (5-HT_1A_) receptor

This receptor is a GPCR expressed in the CNS which is typically coupled to Gi/o which inhibits AC resulting in a decrease in cAMP and neuronal inhibition [109]. It has been reported that CBD is an agonist of this receptor [110],[111]; the other 3 cannabinoids do not appear to have any effect on this receptor.

### 3.9. 5-Hydroxytryptamine (5-HT_3_) receptor

Of the 7 classes of 5-HT receptors, 5-HT_3_ is the only ionotropic cation channel Cys-loop member [112],[113]. Activation results in depolarization mediating fast synaptic transmission [114]. However, unlike the previously discussed nicotine and glycine receptors, 5-HT_3_ receptors are homopentameric so there are no subunit variations [113]. There are 2 alternate 5-HT_3_ transcripts (A and B) but they do not appear to offer any functional differences [115]. CBD, THC, and AEA all appear to directly inhibit 5-HT_3_ receptors when expressed in Xenopus oocytes or HEK-293 cultures [116]–[120]. Fan, as early as 1995, had observed AEA inhibition of these receptors in rat nodose ganglion neurons [121]. Interestingly, it was seen that the degree of inhibition of 5-HT_3_ receptors could vary with the expression system utilized and it has been suggested that this is due to different levels of receptor density at the plasma membrane [122]. 2-AG was not reported as having any effect on this receptor.

### 3.10. Dopamine D2 receptor

This is one of 5 GPCR dopamine receptors found throughout the CNS but especially in the cortex and limbic system; the D2 receptor is coupled to Gi/o and therefore inhibits AC resulting in a decrease in cAMP. Additionally, potassium channels are activated while calcium channels are inhibited resulting in a decrease in neuronal excitability [123]. Seeman has shown that CBD acts directly on D2 receptors where it acts as a partial agonist [124], but it appears there is no other data on the possible effects of the other 3 cannabinoids on this or other dopamine receptors.

### 3.11. Glutamate α-amino-3-hydroxy-5-methyl-4-isoxazolepropionic acid (AMPA) and N-methyl-D-aspartate (NMDA) receptors

Glutamate is the major excitatory transmitter in the human cortex. The 2 principal ionotropic receptor subtypes are the AMPA and NMDA ligand-gated cation selective tetramers that depolarize and, specifically with regards to the NMDA receptor, have a high calcium permeability [125]. CBD negatively allosterically modulates the currents caused by activation of AMPA receptors [126] while AEA inhibits recombinant AMPA receptors expressed in Xenopus oocytes [127]. THC and 2-AG appear to have little direct effect on either type of glutamate receptor. AEA appears to enhance NMDA-induced currents expressed in Xenopus oocytes [128] and in the control of blood pressure in rats [129]. CBD appears not to have been tested on NMDA receptors.

### 3.12. γ-Aminobutyric Acid (GABA_A_) receptors

This pentameric GABA ionotropic chloride channel is the main inhibitory mediator in the CNS [130]. Like glycine, nicotinic, and 5-HT_3_ receptors it is a member of the Cys-loop family of ionotropic ligand-gated channels. So far, 19 GABA_A_ receptor subunit genes have been found in humans which can be assigned to 8 different categories. However, functional channels are normally formed from 2 copies from the α and β categories plus one copy from the others to form a heteropentamer [130],[131]. Koe et al. used synthetic cannabinoid ligands to study GABA_A_ binding, anticonvulsant, and analgesic effects in mice and proposed that cannabinoids might interact with this target [132],[133]. Indeed CBD appears to cause potentiation by acting allosterically with certain α-containing subunits in multiple systems including Xenopus [134]–[136]. In HEK-293 cells expressing this receptor, THC was also shown to potentiate the response [137] while in the same system AEA and 2-AG inhibited currents and increased desensitization of GABA receptors; this was also observed in isolated hippocampal neurons from rat brains [138].

### 3.13. Transient receptor potential (TRP) receptors

TRP receptors compose a large family of cation channels mediating excitation and often calcium entry typically responding to chemical or physical stimuli such as temperature, pressure and pain mediators. They are composed of 4 subunits and can be homo- or heterotetramers and are expressed primarily on sensory neurons [139],[140]. There is much evidence to suggest that many of these receptors are directly affected by cannabinoids to the extent that some in the field have given them the label of ionotropic cannabinoid receptors [7],[141],[142]. On TRPV1-4 receptors, all 4 cannabinoids (CBD, THC, AEA, and 2-AG) appear to act as agonists although the evidence for THC is slightly less compelling [143]–[145]. TRPA1 and TRPM8 are thought to be particularly associated with cold perception [146]. CBD, THC, and AEA all act as agonists on TRPA1 [143],[145],[147] and recently this was also confirmed for 2-AG by Heblinski et al. in HEK cells expressing these channels [148]. Interestingly, the TRPM8 receptor response to the agonists menthol and icilin is antagonized by CBD, THC, and AEA, although there appears to be no published data on the effects of 2-AG [145],[149].

### 3.14. Voltage-gated sodium channels

In most excitable cells the initial depolarizing phase of an action potential is mediated by voltage-gated sodium channels [150]–[152]. These channels typically consist of 3 subunits; a pore-forming α subunit plus 2 β subunits. Nine α subunits (resulting in the classifications of Na_v_1.1 to 1.9) and 4 β subunits have been identified and characterized in mammals [153]. Apart from the so-called gate that opens at the required membrane potential (threshold potential), these channels also inactivate using a second gate to limit excessive depolarization [154],[155]. Using various human and mouse Na_v_1.1 to 1.7 channels, it was found that CBD possibly inhibited these channels by stabilizing the inactivation state and preventing them from opening [156],[157]. THC, although possessing some anticonvulsant properties, does not appear to have similar actions to CBD and due to its psychotropic effects is difficult to study in animal models [158]. However, Turkanis did report inhibition of sodium currents in mouse neuroblastoma cells by THC [159]. Direct effects on these channels by AEA and 2-AG have not been reported.

### 3.15. Voltage-gated calcium channels

For many excitable cells, voltage-gated calcium channels are the primary source of calcium entry, allowing it to act as a secondary messenger for such important functions as neurotransmitter release and muscle contraction [153]. There are 6 classes of voltage-gated calcium channels based on such factors as threshold, pharmacology and rate of inactivation. The classification has now been updated to align with that used for other voltage-gated channels such as L-type calcium channels which are found in many tissues including heart, skeletal muscle and the nervous system that are now called Ca_v_1.1 to 1.4. They are particularly involved in regulating contraction in cardiac and smooth muscle. P and Q-channels are found in the nervous system, smooth muscle, and other tissues and are now classified as Ca_v_2.1 while N and R-channels, also found in the nervous system and other tissues including heart and lung, are now called Ca_v_2.2 and 2.3, respectively. P, Q, and N-channels are involved in neurotransmitter and hormone release. Finally, T-channels that are found in the nervous system, heart, smooth muscle, and other tissues are now classified as Ca_v_3.1–3 and help regulate repolarization in neurons and cardiomyocytes among other functions [160]. These channels are heteropentamers with the main pore-forming subunit α1 typically associating with a β and a γ subunit and additionally α-related gene products such as α2δ [153]. CBD appears to only affect L-type channels/Ca_v_1.1 to 1.4 inhibiting them as shown by Ali et al. in rat ventricular myocytes [161]. Additionally, 2-AG inhibited these channels while THC had no effect [162]. AEA was found to inhibit both T-type channels/Ca_v_3.1–3.3 and L-type/Ca_v_1.1–1.4 [163],[164].

### 3.16. Potassium channels

The potassium channel family is very large and diverse and serves a variety of functions throughout the body, especially in excitable tissues. Repolarization after the sodium-mediated depolarization of the action potential, setting the refractory period/action potential timing and generally opposing excitability when necessary are the main effects of these channels [165],[166]. Classification can generally be sub-divided into 4 smaller families including voltage-gated channels (K_v_1 to 12.3/17 members), calcium or sodium-activated channels (K_Ca_1.1 to 5.1 and K_NA_1.1 and 1.2/8 and 2 members respectively), the so-called two-pore domain or “leak” channels that help establish the resting membrane potential of excitable cells (K_2p_1.1 to 18.1/15 members) and inwardly rectifying channels (K_IR_1.1 to 7.1/16 members) and delayed rectifying channels(K_DR_) [166]. Several fairly recent studies have demonstrated that CBD can enhance the currents of certain voltage-gated potassium channels such as human K_v_7.2 and 7.3 expressed in Chinese hamster ovary cells (CHO) cells, mouse superior cervical ganglion cells and cultured rat hippocampal neurons [167]. Isaev and Topal independently observed inhibition of various potassium delayed rectifier channels and others by CBD using rat, rabbit and dog ventricular myocyte systems [168],[169]. There appears to be little published data on the effects of THC on potassium channels, but back in 1996 Poling et al. published the observation that THC (and AEA) inhibited K_v_1.2 channels in transfected fibroblasts [170]. AEA, apart from enhancing large-conductance K_Ca_1.1 channels in various systems [171] seems to inhibit most potassium channels. Those affected include several cardiac types including human K_v_4.3 and rat myocyte K_ATP_ channels) [172]–[174], K_v_3.1 voltage-gated channels [175], several delayed rectifier channels [176],[177], K_ATP_ (cromakalin-induced) and K_2p_3.1 (TASK-1) channels [178]. Finally, 2-AG was also seen to inhibit several types of potassium channel including Kv4.3 myocyte channels [172] and *I*_A_ current in mouse dopaminergic neurons [179], delayed rectifier channels [177],[180], and K_ATP_ channels in mouse insulinoma cells [180]. The direct actions of the 4 cannabinoids on these targets have been summarized in Table 1.

**Table 1.** A summary of the direct targets of the 4 cannabinoids cannabidiol (CBD), Δ9-tetrahydrocannabinol (THC), N-arachidonoylethanolamine (AEA), and 2-arachidonoylglycerol (2-AG) complete with principal references as discussed in this mini-review. When reporting the effects of these cannabinoids, terminology is used as published. Abbreviations used include (SD) for subunit dependent, N/E for no effect, and N/T for not tested.

**EFFECTOR**	**CBD**	**THC**	**AEA**	**2-AG**	**References**
**CB1R**	-Allosteric	Partial Agonist	Partial Agonist	Partial Agonist	[7],[30],[44]–[52]
**CB2R**	Partial Agonist	Partial Agonist	N/E	Partial Agonist	[7],[30],[44]–[52]
**GPCRs 3,6&12**	Inverse Agonist	N/T	N/T	N/T	[53]–[55]
**GPCR 18**	N/E	Agonist	Agonist	N/E	[58],[59]
**GPCR 55**	Antagonist	Agonist	Partial Agonist	N/E	[60]–[64]
**µ Opioid**	-Allosteric	-Allosteric	N/E	N/E	[66]–[70]
**δ Opioid**	-Allosteric	-Allosteric	N/E	N/E	[70]
**κ Opioid**	N/E	Inhibit	N/E	N/E	[71]
**Adenosine A1,2a**	+Allosteric	N/E	N/E	N/E	[77],[78]
**Adenosine A3**	N/E	N/E	N/E	-Allosteric	[79]
**PPARs**	Agonist	Agonist	Agonist	Agonist	[81]–[91]
**Glycine (SD)**	Potentiate	+Allosteric	+Allosteric	Inhibit	[95]–[99]
**Nicotinic (SD)**	Inhibit	N/E	Antagonist	Antagonist	[101]–[108]
**5-HT_1A_**	Agonist	N/E	N/E	N/E	[110],[111]
**5-HT_3_**	Inhibit	Inhibit	Inhibit	N/E	[116]–[122]
**Dopamine D2**	Partial Agonist	N/T	N/T	N/T	[124]
**NMDA**	N/E	N/E	Enhance	N/E	[128],[129]
**AMPA**	-Allosteric	N/E	Inhibit	N/E	[126],[127]
**GABA_A_ (SD)**	+Allosteric	Potentiate	Inhibit	Inhibit	[132]–[138]
**TRPV1-4**	Agonist	Agonist	Agonist	Agonist	[143]–[145]
**TRPA1**	Agonist	Agonist	Agonist	Agonist	[143],[145],[147],[148]
**TRPM8**	Antagonist	Antagonist	Antagonist	N/T	[145],[149]
**Na_v_1.1-1.7**	Inhibit	Inhibit	N/T	N/T	[156]–[159]
**Ca_v_1.1-1.4**	Inhibit	N/E	Inhibit	Inhibit	[161]–[164]
**Ca_v_3.1-3.3**	N/T	N/T	Inhibit	N/T	[163],[164]
**K_v_7.2-7.3**	Enhance	N/T	N/T	N/T	[167]
**K_DR_**	Inhibit	N/T	Inhibit	Inhibit	[168],[169],[177]–[180]
**K_v_1.2**	N/T	Inhibit	Inhibit	N/T	[170]
**K_Ca_1.1**	N/T	N/T	Enhance	N/T	[171]
**K_v_4.3**	N/T	N/T	Inhibit	Inhibit	[172]–[174]
**K_ATP_**	N/T	N/T	Inhibit	Inhibit	[172],[173],[178],[180]
**K_v_3.1**	N/T	N/T	Inhibit	N/T	[175]
**K_2P_3.1**	N/T	N/T	Inhibit	N/T	[178]

## 4. Future directions

Both the endocannabinoid system and the potential of marijuana phytocannabinoids are now becoming recognized as important areas requiring proper research and appropriate funding. This is finally becoming a reality now that the social stigma associated with marijuana's recreational use is receding. This plant has been used by humans for thousands of years and proper investigation of all its potential uses, contraindications, potential problems with long-term use, etc. need to be fully investigated as its use for medical purposes becomes more widespread. This summary of the actions of 4 principal cannabinoids illustrates the wide array of targets these compounds can affect and underscores why the phytocannabinoids produced by the marijuana plant can have the potential to affect many body systems and disease states. CBD and THC can act as allosteric modulators, both positive and negative, plus other yet to be defined effects on many of these targets. CBD can infact negatively allosterically modulate CB1R itself. AEA and 2-AG have less compelling evidence for allosteric modulation as yet but are still observed to affect many of these targets. Due to the high threshold for inclusion in this mini-review, many papers were not considered as the authors could not state with a high degree of certainty how the cannabinoids were acting. Many publications state cannabinoid-receptor independent but cannot conclude with certainty how the cannabinoid is acting. To overcome this problem, many of the papers included were utilizing fairly simple primary cell cultures expressing specific receptors so they could be certain. It would be nice to see these initial results confirmed in more complex and relevant systems, but this will require the production of very specific synthetic agonists and antagonists to both elucidate mechanisms of action and develop more useful pharmaceuticals. This will also enable further understanding of the function of the endocannabinoid system itself which may reveal a whole new level of subtle modulation of these targets and systems. Hopefully this summary has captured the majority of known direct targets and will help the reader to gain an overall perspective and insight on the functions of these selected cannabinoids and the endocannabinoid system and direct to more specific and detailed resources.
